# PRISM II: an open-label study to assess effectiveness of dextromethorphan/quinidine for pseudobulbar affect in patients with dementia, stroke or traumatic brain injury

**DOI:** 10.1186/s12883-016-0609-0

**Published:** 2016-06-09

**Authors:** Flora M. Hammond, David N. Alexander, Andrew J. Cutler, Stephen D’Amico, Rachelle S. Doody, William Sauve, Richard D. Zorowitz, Charles S. Davis, Paul Shin, Fred Ledon, Charles Yonan, Andrea E. Formella, Joao Siffert

**Affiliations:** Physical Medicine and Rehabilitation, Indiana University School of Medicine, Rehabilitation Hospital of Indiana, 4141 Shore Drive, Indianapolis, IN 46254 USA; University of California, Los Angeles, CA USA; Florida Clinical Research Center, LLC, Bradenton, FL USA; Cornerstone Medical Group, Franklin, TN USA; Baylor College of Medicine, Houston, TX USA; TMS NeuroHealth Centers, Richmond, VA USA; MedStar National Rehabilitation Network, Washington, DC, USA; CSD Biostatistics, Inc., Tucson, AZ USA; Avanir Pharmaceuticals, Inc., Aliso Viejo, CA USA

**Keywords:** Dextromethorphan, Quinidine, Pseudobulbar affect, Dementia, Brain injuries, Stroke, Neuropsychiatric symptoms, Center for neurologic study-lability scale

## Abstract

**Background:**

Phase 3 trials supporting dextromethorphan/quinidine (DM/Q) use as a treatment for pseudobulbar affect (PBA) were conducted in patients with amyotrophic lateral sclerosis (ALS) or multiple sclerosis (MS). The PRISM II study provides additional DM/Q experience with PBA secondary to dementia, stroke, or traumatic brain injury (TBI).

**Methods:**

Participants in this open-label, multicenter, 90-day trial received DM/Q 20/10 mg twice daily. The primary outcome was the Center for Neurologic Study-Lability Scale (CNS-LS), assessing change in PBA episode frequency and severity. The CNS-LS final visit score was compared to baseline (primary analysis) and to the response in a previously conducted placebo-controlled trial with DM/Q in patients with ALS or MS. Secondary outcomes included change in PBA episode count and Clinical Global Impression of Change with respect to PBA as rated by a clinician (CGI-C) and by the patient or caregiver (PGI-C).

**Results:**

The study enrolled 367 participants with PBA secondary to dementia, stroke, or TBI. Mean (standard deviation [SD]) CNS-LS score improved significantly from 20.4 (4.4) at baseline to 12.8 (5.0) at Day 90/Final Visit (change, −7.7 [6.1]; *P* < .001, 95 % CI: −8.4, −7.0). This magnitude of improvement was consistent with DM/Q improvement in the earlier phase-3, placebo-controlled trial (mean [95 % CI] change from baseline, −8.2 [−9.4, −7.0]) and numerically exceeds the improvement seen with placebo in that study (−5.7 [−6.8, −4.7]). Reduction in PBA episode count was 72.3 % at Day 90/Final Visit compared with baseline (*P* < .001). Scores on CGI-C and PGI-C showed that 76.6 and 72.4 % of participants, respectively, were “much” or ”very much” improved with respect to PBA. The most frequently occurring adverse events (AEs) were diarrhea (5.4 %), headache (4.1 %), urinary tract infection (2.7 %), and dizziness (2.5 %); 9.8 % had AEs that led to discontinuation. Serious AEs were reported in 6.3 %; however, none were considered treatment related.

**Conclusions:**

DM/Q was shown to be an effective and well-tolerated treatment for PBA secondary to dementia, stroke, or TBI. The magnitude of PBA improvement was similar to that reported in patients with PBA secondary to ALS or MS, and the adverse event profile was consistent with the known safety profile of DM/Q.

**Trial registration:**

Clinicaltrials.gov, NCT01799941, registered on 25 February 2013

**Electronic supplementary material:**

The online version of this article (doi:10.1186/s12883-016-0609-0) contains supplementary material, which is available to authorized users.

## Background

Affective lability is a commonly described sequela of multiple neurologic disorders and brain injury. Pseudobulbar affect (PBA) is a type of affect lability characterized by sudden, involuntary, and distressing outbursts of laughing and/or crying that are often exaggerated or disconnected from mood state or social context [1–3]. PBA episodes tend to be stereotypical, can last from seconds to several minutes, and often occur multiple times per day. PBA is thought to occur as a result of injury or disease that disrupts pathways regulating emotional expression, or affect, including the corticobulbar tracts and basal ganglia [4, 5]. PBA is diagnosed clinically through a medical and neurologic evaluation and a careful assessment of symptoms in order to distinguish it from features of other psychiatric or neurobehavioral conditions such as depression, anxiety disorders, post-traumatic stress disorder, dementia-related agitation and other conditions that can occur separately or comorbidly with PBA in persons with neurologic diseases or brain injuries [2].

PBA episodes can be highly disruptive to everyday life, causing embarrassment, social isolation and, in some cases, impacting the ability to work [6]. Prevalence estimates vary by rigor of diagnostic requirements, screening methodology, and causative neurological disorder; however a registry sample of 5,290 clinic patients with 1 of 6 neurologic conditions known to be associated with PBA—Alzheimer’s disease (AD), amyotrophic lateral sclerosis (ALS), multiple sclerosis (MS), Parkinson’s disease (PD), stroke, and traumatic brain injury (TBI)— found 36.7 % had a Center for Neurologic Study-Lability Scale (CNS-LS) score ≥13, a score that predicted neurologist diagnosis of PBA in validation studies for 82 % of patients with ALS and 78 % with MS; 9.3 % of this registry sample had a CNS-LS ≥21 [5]. Other studies report PBA symptom prevalence between 7 and 39 % among participants with dementia [5, 7, 8], 5 to 48 % among those with TBI [7, 9, 10], and 11 to 34 % following stroke [11–13]. However, in everyday practice, PBA symptoms are generally under-recognized due to lack of routine screening, limited awareness of the condition and confusion with other neuropsychiatric conditions [3, 7, 14]. Various drugs have been studied as treatments for PBA, including tricyclic antidepressants and selective serotonin reuptake inhibitors, though no agents in these drug classes have been approved for this use [15].

Dextromethorphan hydrobromide and quinidine sulfate in the fixed combination NUEDEXTA® (DM/Q; commercially available since 2010 from Avanir Pharmaceuticals, Inc., Aliso Viejo, CA, USA) is currently the only pharmaceutical agent approved by the US Food and Drug Administration for the treatment of PBA [16]. DM, the CNS-active component of the medication, is a weak, uncompetitive *N*-methyl-d-aspartate (NMDA) receptor antagonist, a sigma-1 receptor agonist, a serotonin and norepinephrine reuptake inhibitor, and an α3β4 neuronal nicotinic receptor antagonist [17–19]. However, the mechanism whereby DM exerts its clinical effects is not fully elucidated. Normally, DM is rapidly metabolized through the cytochrome P450 2D6 (CYP2D6) isoenzyme to dextrorphan, a metabolite that is rapidly glucuronidated, limiting its CNS bioavailability when DM is administered at approved doses. When administered alone, DM achieves only minimal plasma exposure. Low-dose quinidine (10 mg) is a potent inhibitor of cytochrome P450 2D6 that, when combined with DM, substantially increases DM bioavailability and enables greater central nervous system exposure. Well-controlled Phase 3 studies have shown that the DM/Q combination is efficacious for the treatment of PBA [20–22], and is significantly more efficacious than DM alone or Q alone [22].

The DM/Q combination was approved in the United States for the treatment of PBA, irrespective of the type of neurologic etiology, as a result of phase 3 trials in participants with PBA secondary to ALS or MS [20–22]. Across the phase 3 trials in patients with MS or ALS, DM/Q was generally safe and well tolerated [20–22]. In addition, DM/Q was found to be generally well tolerated in a long-term, 52-week safety trial that enrolled participants with PBA associated with a wide range of underlying neurological conditions [23]. To date, there have been only limited clinical trial data evaluating the safety and efficacy of DM/Q for PBA in specific and well-defined patient populations beyond MS and ALS.

The purpose of the present study (referred to as the Pseudobulbar Affect Registry Investigating Symptom Management II [PRISM II] trial) was to provide data on the effectiveness of DM/Q for the treatment of PBA secondary to dementia, stroke, and TBI. This article describes the findings for outcomes common to the overall study population. Outcomes in individual neurologic disease cohorts are reported separately [24–26].

## Methods

### Study design

The study measured effectiveness by evaluating efficacy, safety/tolerability and other patient outcomes when used under usual conditions in patients with dementia, stroke, or TBI. Adults with PBA secondary to dementia, stroke, or TBI and a CNS-LS score ≥13 (scale range, 7 [no symptoms] to 35 [maximum]) were enrolled in an open-label, 90-day study of DM/Q 20/10 mg twice daily (once daily during Week 1). Clinical assessments were performed at baseline, Day 30, and Day 90, with a telephone contact at Day 60. This study was conducted in the United States at 74 enrolling sites, from 26Feb2013 to 30Apr2015, according to Good Clinical Practice and the Declaration of Helsinki. The protocol and study materials were approved by the institutional review board at each site and registered on www.clinicaltrials.gov. (identifier: NCT01799941).

### Participants

Participants were eligible for enrollment if they had a clinical diagnosis of PBA based on published criteria and a clinical diagnosis of dementia, stroke, or TBI [2]. PBA diagnostic criteria were (1) involuntary or exaggerated episodes of emotional expression (i.e., laughing or crying); (2) development of symptoms that represent a change from the person's usual emotional reactivity occurring subsequent to a specified brain disorder; (3) episodes are incongruent with or out of proportion to the individual's mood state and independent to or in excess of a provoking stimulus; and (4) symptoms are not better accounted for by another disorder, substance abuse, or medication use. Patients were also required to have a CNS-LS score ≥13 [27, 28], the same score required for entry into phase 3 trials of DM/Q for PBA [20–22].

Eligible participants had documented diagnoses of one of the following: dementia, (including Alzheimer’s, vascular, Lewy body, or frontotemporal dementia; ischemic or hemorrhagic stroke; or mild, moderate, or severe non-penetrating TBI. There were no restrictions on types of allowed concomitant medications with the exception of those contraindicated by the US Prescribing Information [16], specifically monoamine oxidase inhibitors (MAOIs), and drugs that both significantly prolong QT interval and are primarily metabolized by CYP2D6 (e.g., thioridazine). Other medications typically used by patients with the studied neurologic conditions (including those that are metabolized by CYP2D6) were allowed with the stipulation that medications for management of dementia, such as memantine or acetylcholinesterase inhibitors should be stable for at least 6 weeks and other neuropsychiatric medications such as anticonvulsants, antidepressants, antipsychotics, anxiolytics, and sedative/hypnotics should be stable for at least 2 months prior to baseline; any medication changes, if deemed necessary, were recorded. Potential participants were excluded if they had severe dementia (Mini-Mental State Examination [MMSE] score <10); stroke within 3 months of study enrollment; penetrating TBI; severe depressive disorder; history or current symptoms of schizophrenia (including psychosis), schizoaffective disorder or bipolar disorder; substance/alcohol abuse in the preceding 3 years; systemic disease, neurologic condition or brain injury that was unstable or rapidly changing within the 3 months prior to enrollment; life expectancy ≤6 months; contraindication to DM/Q use (including known QT interval prolongation); DM/Q use during the previous 6 months; or interventional clinical study participation within the preceding 30 days. Written informed consent was obtained from all participants or from legally authorized representatives.

### Outcome measures

#### Primary measure

The primary outcome was the change in CNS-LS score from baseline to Day 90 (or final visit). The CNS-LS was completed by the patient or the caregiver acting as a patient proxy at Day 1 (baseline), Day 30 (visit 1), and Day 90 or early withdrawal (final visit). The CNS-LS is a 7-item (4 laughing items; 3 crying items), self-report rating scale measure of PBA episode frequency and severity that was validated in persons with ALS and persons with MS and is sensitive to change over time and treatment effects [20–22].

#### Secondary measures

**Fig. 1:**
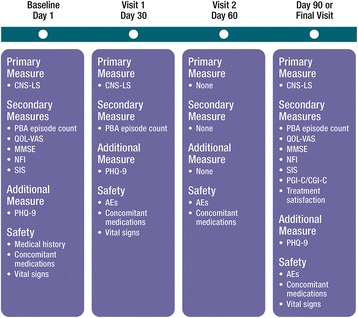
Schedule of study assessments. Caregivers completed ratings as proxies for patients who were unable (except for the MMSE). AE = adverse event; CGI-C = Clinical Global Impression of Change; CNS-LS = Center for Neurologic Study–Lability Scale; MMSE = Mini-Mental State Exam; NFI = Neurobehavioral Functioning Inventory; PBA = pseudobulbar affect; PGI-C = patient/caregiver Global Impression of Change; PHQ-9 = 9-item Patient Health Questionnaire; QOL-VAS = quality-of-life visual analog scale; SIS = Stroke Impact Scale

Secondary measures included PBA episode count for the 7 days preceding each study visit as well as the Clinical Global Impression of Change (CGI-C) and Patient (or Caregiver) Global Impression of Change (PGI-C; 7-point scales ranging from 1 [very much improved] to 7 [very much worse]) based on overall change in the patient’s condition with respect to PBA. In addition, a quality-of-life visual analog scale (QOL-VAS) assessed the degree to which PBA episodes affected the patient’s overall quality of life (11-point scale ranging from 0 [“not at all”] to 10 [“significantly”]) during the past week and a 5-point Likert-type scale rated patient satisfaction with treatment from 1 [very dissatisfied] to 5 [very satisfied]. The Folstein Mini-Mental State Examination (MMSE; 11-item assessment of orientation, memory, attention, and language scored from 0 to 30) was included as a cognitive assessment [29]. As an additional measure, the Patient Health Questionnaire (PHQ-9; a 9-item assessment of depression symptoms with each item rated 0 [not at all] to 3 [nearly every day] based on frequency of occurrence over the past 2 weeks, for a total possible score of 27) was included [30]. Disease-specific functional measures, namely the Neurobehavioral Functioning Inventory and Stroke Impact Scale were assessed in the TBI and stroke cohorts, respectively, and will be reported separately. Caregivers were allowed to complete all assessments except the MMSE for any patients who were unable to do so. The timing of assessments is illustrated in Fig. 1.

#### Safety

Safety measures included reporting of adverse events (AEs) occurring at any time between study enrollment and up to 30 days after the last dose of DM/Q in the study, as well as vital signs and concomitant medication use.

### Statistical analysis

The safety analysis set included all participants who received at least 1 dose of DM/Q. The effectiveness analysis set comprised all participants in the safety set who also met all study eligibility criteria, and completed at least 1 post-baseline CNS-LS assessment. The Medical Dictionary for Regulatory Activities (version 15.1) was used for coding, categorizing, and reporting AEs.

All data were analyzed descriptively. Changes from baseline in ratings at Day 30 and 90 were also analyzed inferentially using one-sample t-tests for rating scale measures (CNS-LS, QOL-VAS, MMSE, and PHQ-9) and a mixed-effects Poisson regression model using number of PBA episodes in the past 7 days as the dependent variable to estimate change in PBA episode counts. The mixed-effects Poisson regression model incorporated age, gender, and time (Day 30 and Day 90) as fixed effects while allowing for individual differences in baseline rate (random-subject effects). The percentage change episode count from baseline to a given visit is 1 minus the appropriate time parameter (λ).

There was no imputation method for missing data; however, if the patient had a Final Visit, that visit was included as the Day 90 Visit. If there was no Final Visit, the Day 30 visit was not carried forward as the subject’s Final Visit.

The primary analysis tested the null hypothesis that the mean change in CNS-LS score from baseline to the Day 90/Final Visit was equal to zero; the 95 % confidence interval (CI) was also reported to enable a descriptive comparison with the CNS-LS change seen in the pivotal phase 3 registration trial (STAR trial) that led to the US approval of DM/Q for PBA [20]. Pearson correlation coefficients were calculated to assess the correlation of CNS-LS scores with other endpoints at baseline, Day 30, and Day 90/Final Visit, as well as correlation of change from baseline in CNS-LS with changes in other measures.

Tests of significance were 2-tailed and carried out at the α = .05 level of significance; all analyses were completed using either SAS v9.2 (SAS Institute Inc., Cary, NC) or Stata v12 (StataCorp, College Station, TX). All patients with data for the given comparison were included.

A power calculation was based on mean (SD) CNS-LS change observed from the pivotal phase 3 trial for the same dose of DM/Q (20/10 mg twice daily): −8.2 (6.1) points vs. placebo: −5.7 (5.3) points. Based on these results, it was determined that a sample size of 100 patients per disease group would provide 80 % power to detect a CNS-LS mean change of −7.45 points (increase of 1.75 points over assumed true placebo mean change of −5.7), or 90 % power to detect a CNS-LS mean change of −7.7 points (increase of 2.0 points over assumed true placebo mean change of −5.7). An interim analysis, conducted after the first 100 patients (regardless of cohort) completed the study, supported these assumptions of magnitude of the effect and indicated that a sample size of 100 per disease group would provide sufficient (≥80 %) power to meet the protocol-specified endpoints. In addition to the results for the entire study population described here, pre-specified analyses for the three distinct diagnosis groups (Dementia, Stroke, and TBI cohorts) were conducted separately and have either been reported in full (Dementia [24]) or have manuscripts in preparation (Stroke [26] and TBI [25]).

## Results

### Patients

**Fig. 2:**
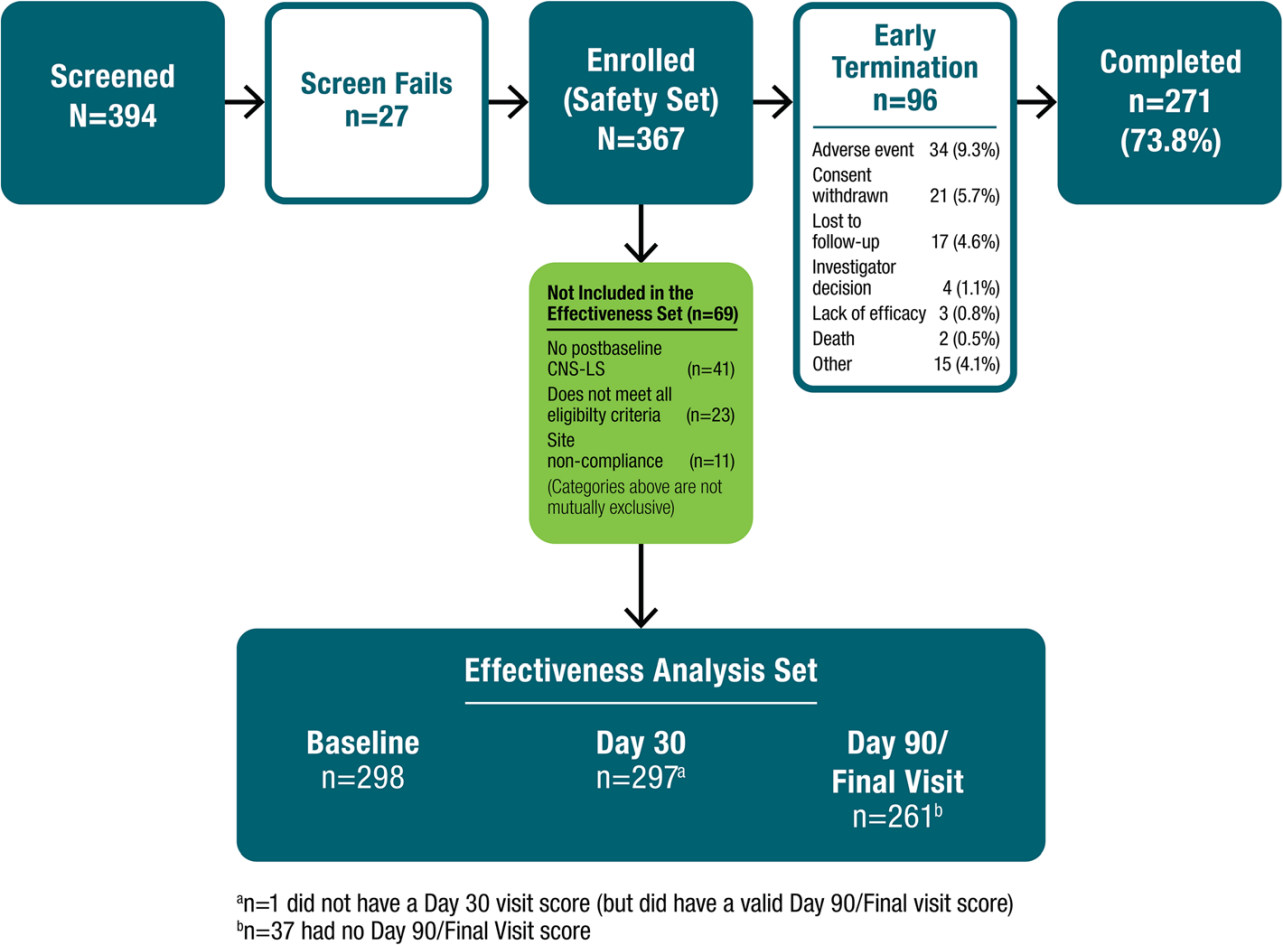
Consort diagram for PRISM II cohort. CNS-LS = center for neurologic study–lability scale

Of the 394 participants screened, 367 participants with PBA (134 with dementia, 113 with stroke, and 120 TBI) were enrolled and received at least 1 dose of DM/Q; 271 (73.8 %) completed the study through Day 90. Participant disposition is shown in Fig. 2; early discontinuation from the study occurred most commonly because of AEs (9.3 %) and withdrawal of consent (5.7 %). Sixty-nine patients (18.8 %) were excluded from the effectiveness analysis set because of one or more of the following: lack of post-baseline CNS-LS score (*n* = 41), failure to meet all study eligibility criteria (*n* = 23), and/or site noncompliance (*n* = 11). A total of 297 patients were included for the Day 30 efficacy analysis, while 261 had evaluable data at Day 90. The baseline demographic and clinical characteristics of the enrolled cohort (safety analysis set) are described in Table 1. The mean (SD) patient age was 59.4 (16.5) years; 17 % lived in a skilled nursing or assisted living facility, and 45 % had a caregiver. In addition, the mean (SD) CNS-LS score at baseline was 20.5 (4.4) and median PBA episode count for the 7 days prior to baseline was 12 (range: 0, 240). Study participants were receiving a median of 7 [0–27] concomitant medications at baseline; the majority (70.8 %) were using least one psychiatric medication, most commonly antidepressants (48.5 %; Table 1). Nearly three quarters (73.0 %) had concomitant cardiovascular disease at baseline, most commonly hypertension (57.8 %) or hyperlipidemia (44.4 %), and the majority (88.6 %) had another central nervous system disorder, most commonly depression (57.5 %) and anxiety disorder (42.2 %).

**Table 1:** Baseline demographics and clinical characteristics—safety analysis set

Characteristic	(*N* = 367)
Age, mean (SD), y	59.4 (16.5)
Age category, n (%)	
≥ 65 years	152 (41.4)
≥ 75 years	75 (20.4)
Gender, n (%)	
Male	165 (45.0)
Female	202 (55.0)
Race, n (%)	
White/Caucasian	304 (82.8)
Black/African American	50 (13.6)
Asian	3 (0.8)
Other^a^	4 (1.1)
Unknown	6 (1.6)
Ethnicity, n (%)	
Hispanic/Latino	71 (19.4)
Presence of a caregiver, n (%)	166 (45.2)
Place of Residence, n (%)	
Home	303 (82.6)
Assisted living	35 (9.5)
Skilled nursing facility	29 (7.9)
Primary diagnosis, n (%)	
Dementia	134 (36.5)
Stroke	113 (30.8)
TBI	120 (32.7)
Concomitant medications at baseline (total no. of medications)	
Mean	7.7
Median (min, max)	7.0 (0, 27)
Psychopharmacologic medication use,^b^ n (%)	260 (70.8)
Any antidepressant	178 (48.5)
SSRIs	103 (28.1)
Other	83 (22.6)
Non-selective (tricyclic)	14 (3.8)
Sedative/hypnotics/anxiolytics	124 (33.8)
Any benzodiazepine^c^	109 (29.7)
Antipsychotics^d^	66 (18.0)
Anticonvulsants	92 (25.1)
CNS-LS score^e,f^	
Mean (SD)	20.5 (4.4)
Median (min, max)	20 (13, 34)
PBA episode count (over 7 days prior to baseline)^f^	
Mean (SD)	21.4 (25.0)
Median (min, max)	12 (0, 240)
MMSE score, mean (SD)^f^	23.9 (5.9)
QOL-VAS, mean (SD)	5.9 (2.6)
PHQ-9,^g^ mean (SD)	13.5 (5.9)

*CNS-LS* Center for neurologic study-lability scale, *MMSE* mini-mental state examination, *PBA* pseudobulbar affect, *PHQ-9* patient health questionnaire-9, *QOL-VAS* quality-of-life visual analog scale, *SD* standard deviation, *SSRI* selective serotonin reuptake inhibitor

^a^Other includes American Indian, Alaskan Native, Native Hawaiian, or other Pacific Islander

^b^Psychopharmacologic medications included anticonvulsants, antipsychotics, antidepressants, sedatives/hypnotics or anxiolytics, and benzodiazepine

^c^Includes benzodiazepines as sedatives/hypnotics plus clonazepam as an anticonvulsant

^d^Typical antipsychotic use in 1.9 %, and atypical antipsychotic use in 16.6 %; categories were not mutually exclusive

^e^The CNS-LS scale ranges from 7 to 35, with higher scores indicating increased frequency and severity of PBA episodes

^f^Effectiveness analysis set (*n* = 298)

^g^PHQ-9 scores range from 0 to 27, with higher scores indicating increased severity of depression

#### Primary efficacy endpoint

**Fig. 3:**
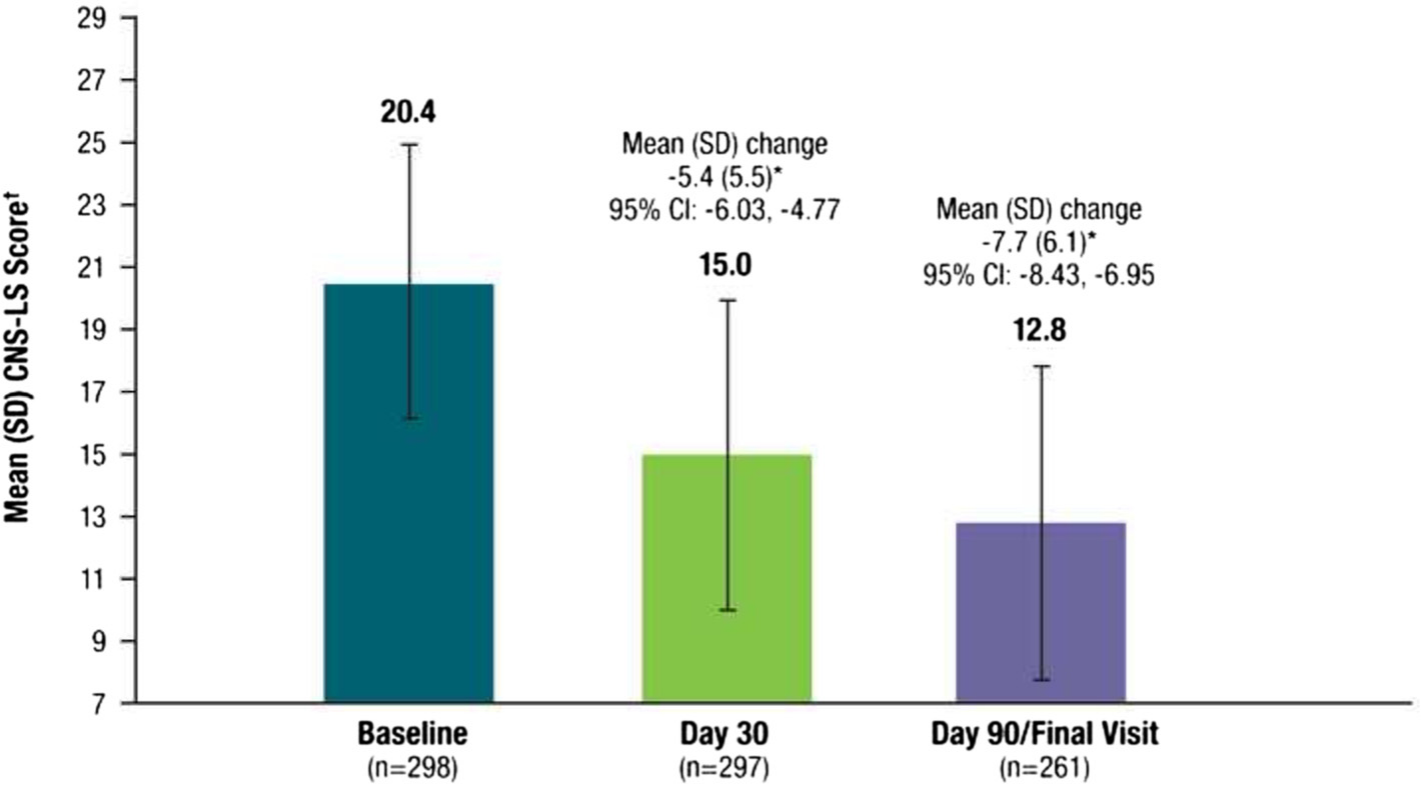
Mean (SD) CNS-LS Score at Baseline, Day 30, and Day 90 (Effectiveness Analysis Set). CNS-LS scores range from 7 to 35, with higher scores indicating increased frequency and severity of PBA episodes. *P* values are based on the one sample t-test and represent comparison with baseline. *P < .001 vs. baseline. ^†^The CNS-LS is a patient-reported quantitative measure of the perceived frequency and severity of PBA episodes; CNS-LS scores were not normalized. CNS-LS = Center for Neurologic Study–Lability Scale; PBA = pseudobulbar affect; SD = standard deviation

Figure 3 depicts the mean (SD) CNS-LS scores at each assessment. Compared with the baseline CNS-LS score, mean (SD) CNS-LS scores at Day 30 (*n* = 297) and Day 90/Final visit (*n* = 261) were 15.0 (5.0) and 12.8 (5.0) and represent a statistically significant improvement from baseline (mean [SD] change at Day 30 of −5.4 [5.5]; 95 % CI: −6.1, −4.8; *P* < .001 and Day 90/Final visit of −7.7 [6.1; 95 % CI: −8.4, −6.9]; *P* < .001). Improvement at Day 90/Final Visit was consistent with that reported for the same dose of DM/Q in the 12-week phase 3 pivotal trial that enrolled patients with PBA secondary to ALS or MS (mean [95 % CI] change from baseline, −8.2 [−9.4, −7.0]) and represents an improvement over the placebo group from that study (−5.7 [−6.8, −4.7]).

#### Secondary analyses

**Fig. 4:**
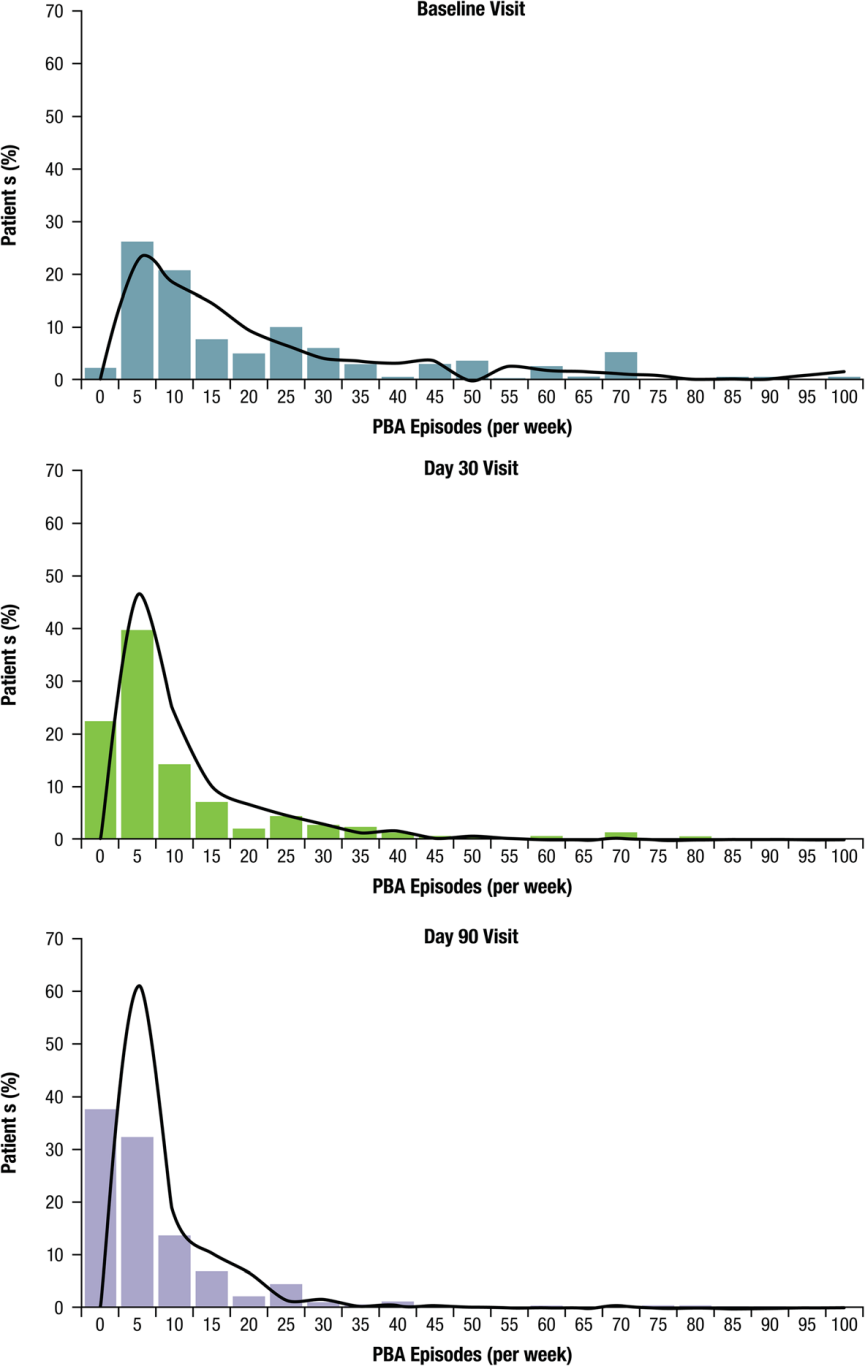
Distribution of PBA Episodes (occurring in the past 7 days) by Visit. Solid bars illustrate the percentage of patients experiencing the given number of episodes shown within the range provided on the x axis. The solid curved line represents the number of PBA episodes that would be predicted based on each patient’s values for the parameters (age, gender and time [Day 30, Day 90]; fixed effects) and baseline rate (random effects) in the mixed-effects Poisson regression model. Patients or daytime caregivers were asked to identify, count, and recall the total episodes of exaggerated or uncontrollable laughing and/or crying over the previous 7 days (prior to visit) at baseline, Day 30, and Day 90. Estimated percent change from baseline for PBA episode count was evaluated via a mixed-effects Poisson regression model for the effectiveness analysis set. *P < .001 vs. baseline

PBA episode counts over the 7 days prior to study visit decreased from a median of 12 at baseline to 4 at Day 30 and 2 at Day 90. PBA episode frequency by study visit is shown in Fig. 4, using the mixed-effects Poisson model estimates. PBA episodes were reduced overall by an estimated 57.5 % at Day 30 and 72.3 % at Day 90 compared with baseline (*P* < .001 for both). Remission of PBA episodes (defined as no reported episodes in the week before assessment) was reported by 20.3 % of the sample at Day 30 and 35.4 % at Day 90.

**Fig. 5:**
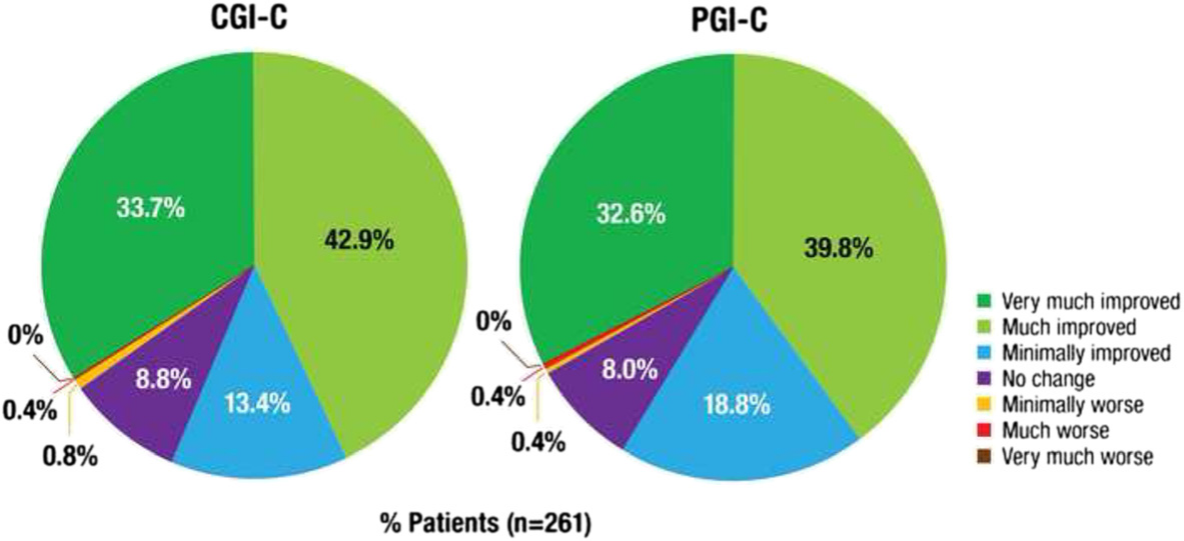
90-Day Clinical and Patient Global Impression of Change (Effectiveness Analysis Set). CGI-C is a 7-point investigator-rated scale that assessed overall treatment response (with respect to PBA) from baseline to Day 90/Final Visit, rated as very much improved, much improved, minimally improved, no change, minimally worse, much worse, or very much worse. PGI-C is a 7-point patient/patient’s caregiver rated scale that assessed overall treatment response (with respect to PBA) from baseline to Day 90/Final Visit, rated as very much improved, much improved, minimally improved, no change, minimally worse, much worse, or very much worse. CGI-C = Clinical Global Impression of Change; PGI-C = Patient/Caregiver Global Impression of Change

Global ratings by clinicians and participants/caregivers indicated the majority believed that during study treatment there had been substantial overall improvement with respect to PBA (Fig. 5). At Day 90/Final Visit, 76.6 % of patients were rated by their clinician as “very much improved” or “much improved” on CGI-C and 72.4 % of participants (or caregiver proxies) rated themselves as “very much improved” or “much improved” with respect to PBA compared with baseline. In contrast, 2 patients reported being “minimally worse” or “much worse” on PGIC (0.8 %) and 3 were reported to be “minimally” or “much worse” on CGIC (1.1 %). No patient was reported as “very much worse” on either measure. When asked about satisfaction with the treatment, 47.5 % of participants (or caregivers) indicated they were very satisfied, 28.0 % somewhat satisfied, 11.5 % neutral, 5.4 % somewhat dissatisfied, and 7.7 % very dissatisfied.

The mean QOL-VAS rating of PBA episode impact on quality of life, the PHQ-9 depressive symptom measure, and the MMSE cognitive measure all improved during the study. QOL-VAS scores improved from a mean (SD) of 5.9 (2.6) at baseline to 2.7 (2.6) at Day 90/Final Visit (mean [SD] change from baseline, −3.1 [3.2]; *P* < .001). PHQ-9 scores improved from mean (SD) 13.5 (5.9) at baseline (moderate depression) to 7.5 (5.5) at Day 90/Final Visit (mild depression), representing a mean [SD] change from baseline of −5.6 [6.2] points (*P* < .001)); mean (SD) MMSE scores increased from 23.9 (5.9) at baseline to 24.3 (6.0) at Day 90/Final Visit (mean change from baseline, 0.60 (3.0); *P* < .01). For the correlation analysis, CNS-LS score reduction from baseline to Day 90 was significantly correlated with improvements in all secondary outcomes (weekly PBA episode rate, QOL-VAS, PGI-C, CGI-C, PHQ-9, and treatment satisfaction; all *P* < .001) except change in MMSE (Table 2).

**Table 2:** Correlation of change from baseline to day 90/final visit in CNS-LS score with other outcome measures—effectiveness analysis Set

Variable	Pearson Correlation	*P* value
Weekly PBA episode rate change	0.21	<.001
QOL-VAS change	0.40	<.001
PGI-C	0.47	<.001
CGI-C	0.48	<.001
PHQ-9 change	0.32	<.001
Patient satisfaction score	−0.22	<.001
MMSE change	0.01	.877

*CGI-C* clinical global impression of changes, *CNS-LS* Center for neurologic study—lability scale, *MMSE* mini-mental state exam, *PBA* pseudobulbar affect, *PGI-C* patient/caregiver global impression of change, *PHQ-9* 9-item patient health questionnaire, *QOL-VAS* quality-of-life visual analog scale

### Safety

AEs are summarized in Table 3. Of the 367 participants who received DM/Q and comprised the safety set, 132 (36.0 %) reported at least 1 AE, including the 55 (15.0 %) who reported at least 1 AE deemed at least possibly related to DM/Q. The most frequently reported AEs were diarrhea (5.4 %), headache (4.1 %), urinary tract infection (2.7 %), and dizziness (2.5 %). Most AEs were of mild or moderate intensity; severe AEs occurred in 6.0 % of participants. In total, 36 (9.8 %) participants had AEs that led to study withdrawal, most commonly for diarrhea (8 [2.2 %]), dizziness, affective lability, and agitation (3 [0.8 %] each. Twenty-three (6.3 %) participants experienced a serious AE; no serious AE was considered treatment related by the study investigators. Two deaths occurred during the study (two males ages 91 and 83 years with dementia); both were deemed not related to study drug after careful review by the investigators and were reported in the dementia cohort manuscript [24].

**Table 3:** Summary of adverse events—safety analysis set

AE Summary, n (%)	(*N* = 367)
Any AE	132 (36.0)
AE intensity	
Mild	67 (18.3)
Moderate	72 (19.6)
Severe	22 (6.0)
Unknown	7 (1.9)
Treatment-related AEs	55 (15.0)
Treatment-related AE intensity	
Mild	23 (6.3)
Moderate	28 (7.6)
Severe	7 (1.9)
Unknown	3 (0.8)
Serious AEs	23 (6.3)
Treatment-related serious AEs	0
AEs leading to discontinuation	36 (9.8)
Frequency of AEs by preferred term (occurring in >1 % of patients)	
Diarrhea	20 (5.4)
Headache	15 (4.1)
Urinary tract infection	10 (2.7)
Dizziness	9 (2.5)
Nausea	6 (1.6)
Fall	6 (1.6)
Fatigue	5 (1.4)
Somnolence	5 (1.4)
Dry mouth	4 (1.1)
Gastroesophageal reflux disease	4 (1.1)
Agitation	4 (1.1)
Peripheral edema	4 (1.1)

*AE* adverse event

## Discussion

Earlier placebo-controlled trials performed with DM/Q included persons with PBA secondary to MS or ALS. The PRISM II open-label, 90-day trial provides expanded safety and efficacy data for DM/Q in a broader patient population and is the first prospectively conducted, systematic study to evaluate DM/Q effectiveness for PBA occurring subsequent to dementia, stroke, or TBI. The inclusion criteria defined within this trial allowed for a patient population that more closely resembles “real-life” clinical circumstances (e.g., participants in this trial were taking a median of 7 concomitant medications, about half were on antidepressants and nearly 20 % were in an assisted living or skilled nursing facility).

Substantial improvement in clinical symptoms of PBA were seen at both post-baseline assessments with a −7.7 point (SD 6.1) reduction in CNS-LS score and a 72.3 % reduction in PBA episode frequency at Day 90/Final visit, both of which represented statistically significant differences compared with baseline. Improvements were consistent across the three cohorts including patients with stroke, dementia and TBI. The mean (12.8) CNS-LS score at study endpoint was below the minimum threshold required for study inclusion. Since this study allowed caregivers to complete assessments for patients who were unable to do so, the planned statistical analyses allowed for an evaluation of caregiver-completed vs. patient-completed outcomes. This was done for the dementia cohort, and as previously described, caregivers generally reported greater PBA symptom change than patients, but between group differences were only statistically significant (*P* < .001) for the estimated PBA episode count reduction (57.7 % for patient-reported vs. 77.2 % for caregiver-reported ratings) [24].

Improvement was also observed in all other secondary endpoints, including a clinical meaningful reduction in PHQ-9 scores (minimal clinically important difference for the PHQ-9 has been estimated to be a 5 point change [31]; mean change observed in this trial was −5.6). In addition, DM/Q was well tolerated, with 9.8 % of participants having AEs leading to discontinuation; the most frequent AEs were diarrhea (5.4 %), headache (4.1 %), urinary tract infection (2.7 %), and dizziness (2.5 %).

### Clinical implications

Although the present study was open label, results appear to be clinically meaningful, consistent across study cohorts, and consistent with those seen in well-controlled, phase 3 trials of DM/Q. The clinical relevance of CNS-LS score reductions from baseline to Day 90/Final Visit is reflected in corresponding reductions in PBA episodes and improvements on other secondary measures, including clinician and patient global impressions with respect to PBA and satisfaction with treatment. CNS-LS reduction was moderately but significantly correlated with improvements in all secondary outcomes (weekly PBA episode rate, QOL-VAS, PGI-C, CGI-C, PHQ-9, and treatment satisfaction; all *P* < .001) with the exception of the MMSE. While the measured outcomes are directionally related, the lack of strong correlation (all Pearson’s coefficients were < .5) suggests that changes in CNS-LS and other outcomes are not solely reflective of changes in PBA episode number and may reflect other aspects of PBA episodes, such as episode intensity, subjective perception, functioning related to these episodes, or other factors not readily apparent from these data.

**Fig. 6:**
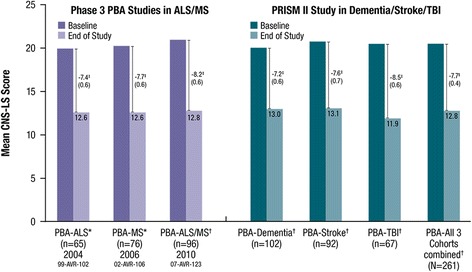
Mean CNS-LS Scores Across DM/Q Studies for PBA Secondary to Diverse Neurologic Conditions. *DM/Q 30/30 mg twice daily; †DM/Q 20/10 mg twice daily. ‡Improvement from baseline in mean CNS-LS (SE). 99-AVR-102 (4 week study comparing DM/Q to DM or Q monotherapy): End of study is the mean of the CNS-LS scores for Days 15 and 29; *P* = 0.001 vs. dextromethorphan comparator and *P* < 0.001 vs quinidine comparator. 02-AVR-106 (12 week DBPC study): End of study is the mean of the CNS-LS scores on Days 15, 29, 57, and 85; *P* < 0.0001 vs. placebo. 07-AVR-123 (12 week DBPC study): End of study is at Week 12 intent to treat; *P* < 0.05 vs. placebo. PRISM II: End of study is at Day 90/Final Visit; *P* < 0.001 vs. baseline in all 3 cohorts. ALS = amyotrophic lateral sclerosis; CNS-LS = Center for Neurologic Study–Lability Scale; DM/Q = dextromethorphan/quinidine; MS = multiple sclerosis; PBA = pseudobulbar affect; TBI = traumatic brain injury; SE = standard error

A pre-specified analysis for this trial assessed the 95 % CI for mean change from baseline in CNS-LS scores in order to enable descriptive comparisons with the earlier placebo-controlled Phase 3 trial in patients with PBA secondary to ALS or MS [20]. Although drug performance cannot be directly compared across different clinical trial populations and settings, PBA symptom improvement with DM/Q in the current open-label trial is consistent with what was observed with DM/Q in the pivotal Phase 3 trial published by Pioro et al. [20], with a magnitude of improvement larger than observed for the group randomized to placebo in that study. These findings are also consistent with two other earlier Phase 3 randomized, controlled trials examining the effect of DM/Q for PBA in patients with MS or ALS (range, 7.4–7.7; Fig. 6). CNS-LS reductions were similar across all 3 disease cohorts included in this PRISM II trial (dementia, stroke, and TBI; range, 7.2–8.5; Fig. 6) [24–26]. Taken together, data from PRISM II expand on prior observations of the utility of DM/Q for PBA in MS and ALS and provide consistent and clinically meaningful evidence of DM/Q effectiveness for PBA in patients with 3 additional common, and disparate neurological etiologies, namely dementia, TBI and stroke.

DM/Q was associated with low incidence of overall AEs (36 %), treatment-related AEs (15 %), and AEs leading to withdrawal (9.8 %). Commonly encountered AEs were consistent with the safety data from controlled studies with DM/Q and information included in the approved product label; no new safety signals emerged in this clinical trial, and none of the reported serious AEs (6.3 %) were deemed by investigators to be treatment related.

Limitations of this study are primarily related to its open-label design without an active or placebo comparator group. In addition, use of self-reported measures may be susceptible to subjective bias based on observations by patient, caregiver, or investigator. Lastly, the criteria for the effectiveness analysis population that required patients to meet all exclusion and inclusion criteria may have introduced bias.

The CNS-LS is validated as a predictive screening assessment for PBA episode frequency and severity based on studies in patients with ALS and MS; [21, 22] however, this scale has not been specifically validated for use in screening in patient populations with dementia, stroke, and TBI. Even so, changes in CNS-LS in this study were consistent with improvements in several other measures of clinical relevance for PBA (e.g. frequency of PBA episodes), and prior epidemiological studies have shown that CNS-LS scores increase with perceived PBA episode burden [5].

## Conclusions

Study findings showed that DM/Q 20/10 mg administered twice daily in non-blinded fashion over 12 weeks to participants with PBA secondary to dementia, stroke, or TBI was generally well tolerated and was associated with improvements in CNS-LS scores and reductions in PBA episodes. These observations are consistent with those seen in the well-controlled phase 3 trials of DM/Q conducted in those with PBA secondary to ALS or MS. Improvement in PBA symptoms was associated with clinically meaningful improvements in CGI-C and PGI-C ratings with respect to PBA. The effectiveness of DM/Q for PBA secondary to these conditions is consistent with the proposed pathophysiology of PBA, which suggests symptoms arise when there is disruption or damage to brain pathways regulating emotional expression, regardless of the specific underlying neurologic condition.

## Abbreviations

AD, Alzheimer’s disease; AE, adverse event; ALS, amyotrophic lateral sclerosis; CGI-C, Clinical Global Impression of Change; CI, confidence interval; CNS-LS, Center for Neurologic Study–Lability Scale; CYP2D6, cytochrome P450 2D6; DM/Q, dextromethorphan/quinidine; MMSE, Mini-Mental State Examination; MS, multiple sclerosis; NFI, neurobehavioral functioning inventory; NMDA, *N*-methyl-d-aspartate; PBA, pseudobulbar affect; PD, Parkinson’s disease; PGI-C, Patient/Caregiver Global impression of change; PHQ-9, patient health questionnaire-9; PRISM, Pseudobulbar affect registry investigating symptom management; QOL-VAS, quality-of-life visual analog scale; SD, standard deviation; SE, standard error; SIS, stroke impact scale; SSRI, selective serotonin reuptake inhibitor; TBI, traumatic brain injury.

## Additional files

Additional file 1: Institutional Review Boards. File contains a listing location and members of Institutional Review Boards approving the study protocol. (PDF 30 kb)
