# Erratum to: PRISM II: an open-label study to assess effectiveness of dextromethorphan/quinidine for pseudobulbar affect in patients with dementia, stroke or traumatic brain injury

**DOI:** 10.1186/s12883-016-0679-z

**Published:** 2016-09-02

**Authors:** Flora M. Hammond, David N. Alexander, Andrew J. Cutler, Stephen D’Amico, Rachelle S. Doody, William Sauve, Richard D. Zorowitz, Charles S. Davis, Paul Shin, Fred Ledon, Charles Yonan, Andrea E. Formella, Joao Siffert

**Affiliations:** 1 Physical Medicine and Rehabilitation, Indiana University School of Medicine, Rehabilitation Hospital of Indiana, 4141 Shore Drive, Indianapolis, IN 46254 USA; 2 University of California, Los Angeles, CA USA; 3 Florida Clinical Research Center, LLC, Bradenton, FL USA; 4 Cornerstone Medical Group, Franklin, TN USA; 5 Baylor College of Medicine, Houston, TX USA; 6 TMS NeuroHealth Centers, Richmond, VA USA; 7 MedStar National Rehabilitation Network, Washington, DC USA; 8 CSD Biostatistics, Inc., Tucson, AZ USA; 9 Avanir Pharmaceuticals, Inc., Aliso Viejo, CA USA

## Erratum

After publication of the original article [1], the authors noticed that there were errors in the caption of Fig. 3, and the y-axis of Fig. 6 itself.

**Fig. 6:**
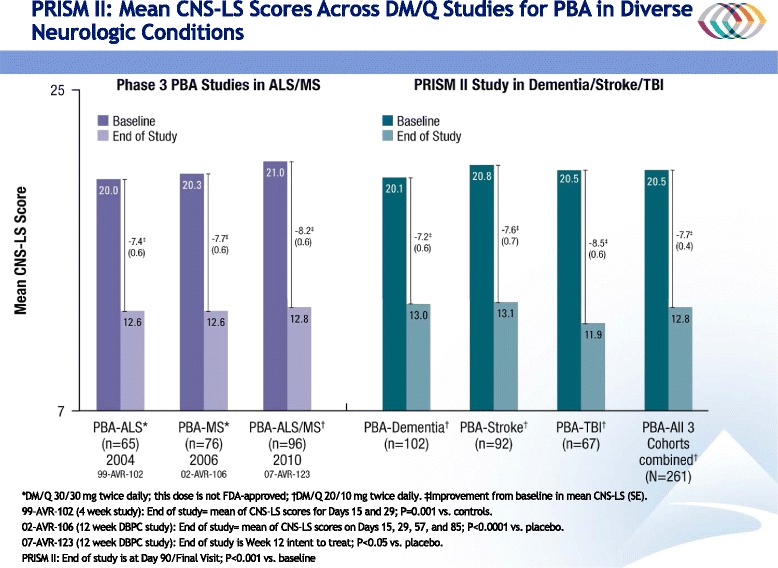
Mean CNS-LS Scores Across DM/Q Studies for PBA Secondary to Diverse Neurologic Conditions. *DM/Q 30/30 mg twice daily; †DM/Q 20/10 mg twice daily. ‡Improvement from baseline in mean CNS-LS (SE). 99-AVR-102 (4 week study comparing DM/Q to DM or Q monotherapy): End of study is the mean of the CNS-LS scores for Days 15 and 29; *P* = 0.001 vs. dextromethorphan comparator and *P* < 0.001 vs quinidine comparator. 02-AVR-106 (12 week DBPC study): End of study is the mean of the CNS-LS scores on Days 15, 29, 57, and 85; *P* < 0.0001 vs. placebo. 07-AVR-123 (12 week DBPC study): End of study is at Week 12 intent to treat; *P* < 0.05 vs. placebo. PRISM II: End of study is at Day 90/Final Visit; *P* < 0.001 vs. baseline in all 3 cohorts. ALS = amyotrophic lateral sclerosis; CNS-LS = Center for Neurologic Study–Lability Scale; DM/Q = dextromethorphan/quinidine; MS = multiple sclerosis; PBA = pseudobulbar affect; TBI = traumatic brain injury; SE = standard error

The following statement should not have been included in the caption of Fig. 3: “CNS-LS scores were not normalized.” The CNS-LS is a rank-order scale, and is not normalized. This statement was included erroneously and the authors intended on removing it prior to resubmission, but this was unfortunately overlooked.

Similarly, the y-axis within Fig. 6 was mislabelled. The CNS-LS scale ranges from 7 to 35, so the y-axis for Fig. 6 should start at a base score of 7 and not zero. The correct and updated version of Fig. 6, in which the data presented remain accurate and are unchanged, is published in this erratum.
