# Supplementary material for: PRISM II: an open-label study to assess effectiveness of dextromethorphan/quinidine for pseudobulbar affect in patients with dementia, stroke or traumatic brain injury
Source: BMC Neurol. 2016 Jun 9;16:89. doi: 10.1186/s12883-016-0609-0 (PMC4899919; doi:10.1186/s12883-016-0609-0)
Supplement: Additional file 1: — Institutional Review Boards. File contains a listing location and members of Institutional Review Boards approving the study protocol. (PDF 30 kb) [file 12883_2016_609_MOESM1_ESM.pdf]

16.1.3 List of IECs or IRBs and Sample Consent Form(s)

**IEC/IRB**	**Investigator (Number)**
Copernicus Group IRB<br>P.O. Box 110605<br>Research Triangle Park, NC 27709, USA	Acevedo, Paul A (257)<br>Ahmed, Syed Shoaib (101)<br>Ajtai, Bela (167)<br>Akhtar, Nadeem (199)<br>Akmal Shamsi, Mohammad Ali (235)<br>Allison, Dale Crawford (520)<br>Alva, Gustavo (106)<br>Alvarez, Maria Victoria (156)<br>Anderson, Allan A. (258)<br>Antin, Todd M (107)<br>Arends, Douglas (168)<br>Arnold, Thomas W (157)<br>Arocha, Emelina A (216)<br>Axley, John H (108)<br>Baber, Riaz Ahmad (238)<br>Balter, Jordan M (190)<br>Barker, James T (538)<br>Bayrakdarian, Carlo (102)<br>Benjamin, Sabrina Ann (225)<br>Berger, Malcolm (229)<br>Blake, David M (256)<br>Blanco, Guillermo (542)<br>Booker, J Gary (217)<br>Bowers, David N (263)<br>Brar, Saroj (110)<br>Brenes, Joseph A (111)<br>Bull, Janet (206)<br>Capps, Raymond L (237)<br>Carey, John Timothy (218)<br>Chappel, Christopher M (115)<br>Chepuru, Yadagiri (173)<br>Choi, John Y (118)<br>Collins, John O (103)<br>Crane, Monica (120)<br>Crumpacker, David W (121)<br>Cusi, Antonio (201)<br>D'Amico, Stephen J (202)<br>De Bien, Laura (540)<br>De Llanos, Ariel (264)<br>Desai, Urvi (191)<br>Dreize, Richard M (519)<br>Fellus, Jonathan (122)<br>Ferencz, Gerald J (219)<br>Fruchter, Gerald (558)<br>Fullerton, John H (126)<br>Genovese, Michael V (251)<br>Gheorghiu, Bogdan P (174)

IEC/IRB	Investigator (Number)
	Goldberg, Sheldon (128)<br>Goldstein, Susanna (129)<br>Gonzalez, Blanca (211)<br>Goodwin, John Joseph (196)<br>Green, Benny (545)<br>Gudesblatt, Mark (537)<br>Harding, Herndon Price (261)<br>Horst, James (207)<br>Iqbal, Naveed (180)<br>Isaacson, Stuart H (503)<br>Kanneganti, Prasad (200)<br>Kellogg, Jason (132)<br>Kellogg, Kevin (228)<br>Kim, Lance (133)<br>Knubley, William Arthur (134)<br>Kohlenberg, Cary J (135)<br>Li, George (534)<br>Linden, David E (136)<br>Lopez, Jose C (249)<br>Mankowski, Kenneth A (234)<br>Martinez, Kenneth Patrick (224)<br>McLaughlin, Paul E (527)<br>Michel, Elliot M (248)<br>Moore, Norman (141)<br>Moya, Jaynier (536)<br>Pardo, Ricardo R (146)<br>Pass, Mark D (241)<br>Patel, Meenakshi (147)<br>Pfeffer, Michael M (528, 546)<br>Pretorius, Harold T (255)<br>Pulver, David C (539)<br>Quinn, Pamela Michele Galvin (250)<br>Raikhel, Marina (528)<br>Ranjan, Rakesh (259)<br>Richard, John W (246)<br>Rios, Jose A (253)<br>Riser, John Byron (149)<br>Rodriguez, Ivan D (253)<br>Ross, Daniel (104)<br>Roudachevski, Evgueni (240)<br>Rubin, Bruce (165, 267)<br>Sanchez, Sigrid A (528)<br>Schechter, Steven H (533)<br>Serentill, Luis (506)<br>Shafer, Stuart James (510)<br>Shua-Haim, Joshua R (150)<br>Singh, Upinder (166)<br>Sky, Adam (151)<br>Smirnoff, Alexander John (188)

**IEC/IRB**	**Investigator (Number)**
	Stalker, Andrew P (232)<br>Stoltz, Steven M (513)<br>Tobias, Hal M (153)<br>Vaishnavi, Sandeep (209)<br>Valdes, Martin (221)<br>Vasquez, Alberto B (509)<br>Vidic, Thomas R (154)<br>Walton, Marie L (529)<br>Weinstein, Bryan Sidney (195)<br>Wellman, Charles V (222)<br>Yapundich, Robert A (505)<br>Zarate-Rowell, Elizabeth (544)
Wayne State University Institutional Review Board<br>IRB Administration Office<br>87 East Canfield, Second Floor<br>Detroit, MI 48201, USA	Meythaler, Jay (223)
Wake Forest University Health Sciences<br>Office of Research<br>Institutional Review Board<br>Medical Center Boulevard<br>Winstom-Salem, NC 27157-1023, USA	Siddiqui, Mustafa (198)
The State University of New York at Buffalo Health Sciences Institutional Review Board<br>Clinical Translational Research Center<br>875 Ellicot Street, Room 5018<br>Buffalo, NY 14203, USA	Ching, Marilou (117)
Atlantic Health System Institutional Review Board<br>475 South Street<br>Morristown, NJ 07960, USA	Felberg, Robert A (210)
Burke Rehabilitation Hospital Committee for Human Rights in Research<br>785 Mamaroneck Avenue<br>White Plains, NY 10605, USA	Fonzetti, Pasquale (124)
University of Louisville Institutional Review Board<br>MedCenter One, Suite 200<br>501 E. Broadway<br>Louisville, KY 40202-1798, USA	Kaelin, Darryl (193)
Baylor Research Institute IRB<br>3310 Live Oak, Suite 501<br>Dallas, TX 75204	Khoury, Chaouki (215)

IEC/IRB	Investigator (Number)
Vanderbilt University Institutional Review Board<br>504 Oxford House<br>Nashville, TN 37232-4315, USA	Kirshner, Howard S. (245)
Western Institutional Review Board<br>1019 39th Avenue, SE, Suite 120<br>Puyallup, WA 98374-2115, USA	Lippa, Carol (137)<br>Jacome, Tomas H (197)
Poudre Valley Health System Institutional Review Board<br>2002 Caribou, Suite 100<br>Fort Collins, CO 80525, USA	McIntosh, Gerald C. (254)
East Texas Medical Center Regional Healthcare System Institutional Review Board<br>721 Clinic Drive<br>Tyler, TX 75701, USA	Plotkin, George (247)
Institutional Review Board for Human Research at St. Joseph's Hospital and Medical Center<br>Research Administration<br>350 West Thomas Road<br>Phoenix, AZ 85013, USA	Shi, Jiong (220)
The Methodist Hospital Research Institute<br>6670 Bertner Street<br>Houston, TX 77030, USA	Spann, Bryan (152)
